# Health Is a Duty

**Published:** 1880-08

**Authors:** 


### HEALTH IS A DUTY.

"A dying man can do nothing easy," were the last words of the immortal Franklin. A diseased man can do nothing well, are words of our own, quite as true.

If any thing should be well done, it should be the preparation which is needed to fit us for the exalted condition which has just been described, and to do it well, the highest health and the longest life should be sought by all. Such a preparation should be made under the most favorable of all possible conditions, and it is to no less an end, that this book has been conceived, to wit, to show the reader how health may be maintained, and how disease may be averted to the utmost limit of human life, that by the aid of health and length of days, the most perfect preparation possible may be made for the immortal existence beyond, and in this light, who shall deny that Health is a duty? Echo answers, 'Disease is a crime!'

"*Health and Disease.*"

